# Calcium-Alginate-Chitosan Nanoparticle as a Potential Solution for Pesticide Removal, a Computational Approach

**DOI:** 10.3390/polym15143020

**Published:** 2023-07-12

**Authors:** Osvaldo Yáñez, Melissa Alegría-Arcos, Reynier Suardiaz, Luis Morales-Quintana, Ricardo I. Castro, Jonathan Palma-Olate, Christian Galarza, Ángel Catagua-González, Víctor Rojas-Pérez, Gabriela Urra, Erix W. Hernández-Rodríguez, Daniel Bustos

**Affiliations:** 1 Núcleo de Investigación en Data Science, Facultad de Ingeniería y Negocios, Universidad de las Américas, Santiago 7500000, Chile; 2 Departamento de Química Física, Facultad de Ciencias Químicas, Universidad Complutense de Madrid, 28040 Madrid, Spain; reysuard@ucm.es; 3 Multidisciplinary Agroindustry Research Laboratory, Facultad de Ciencias de la Salud, Universidad Autónoma de Chile, Talca 3400000, Chile; luis.morales@uautonoma.cl; 4 Multidisciplinary Agroindustry Research Laboratory, Carrera de Ingeniería en Construcción, Instituto de Ciencias Químicas Aplicadas, Universidad Autónoma de Chile, Talca 3400000, Chile; ricardo.castro@uautonoma.cl; 5 Facultad de Ingeniería, Universidad de Talca, Curicó 3340000, Maule, Chile; jonathan.palma@utalca.cl; 6 Escuela Superior Politécnica del Litoral, Guayaquil EC090903, Ecuador; chedgala@espol.edu.ec (C.G.); anglucat@espol.edu.ec (Á.C.-G.); 7 Doctorado en Biotecnología Traslacional, Facultad de Ciencias Agrarias y Forestales, Universidad Católica del Maule, Talca 3480094, Chile; victor.rojas.03@alu.ucm.cl; 8 Laboratorio de Bioinformática y Química Computacional, Departamento de Medicina Traslacional, Facultad de Medicina, Universidad Católica del Maule, Talca 3480094, Chile; gabriela.urra@alu.ucm.cl; 9 Unidad de Bioinformática Clínica, Centro Oncológico, Facultad de Medicina, Universidad Católica del Maule, Talca 3480094, Chile; 10 Centro de Investigación de Estudios Avanzados del Maule (CIEAM), Vicerrectoría de Investigación y Postgrado Universidad Católica del Maule, Talca 3460000, Chile

**Keywords:** virtual screening, ensemble-docking, molecular dynamics simulation, alginate-chitosan, nanoparticle, polyelectrolyte matrix, pesticide adsorption

## Abstract

Pesticides have a significant negative impact on the environment, non-target organisms, and human health. To address these issues, sustainable pest management practices and government regulations are necessary. However, biotechnology can provide additional solutions, such as the use of polyelectrolyte complexes to encapsulate and remove pesticides from water sources. We introduce a computational methodology to evaluate the capture capabilities of Calcium-Alginate-Chitosan (CAC) nanoparticles for a broad range of pesticides. By employing ensemble-docking and molecular dynamics simulations, we investigate the intermolecular interactions and absorption/adsorption characteristics between the CAC nanoparticles and selected pesticides. Our findings reveal that charged pesticide molecules exhibit more than double capture rates compared to neutral counterparts, owing to their stronger affinity for the CAC nanoparticles. Non-covalent interactions, such as van der Waals forces, π-π stacking, and hydrogen bonds, are identified as key factors which stabilized the capture and physisorption of pesticides. Density profile analysis confirms the localization of pesticides adsorbed onto the surface or absorbed into the polymer matrix, depending on their chemical nature. The mobility and diffusion behavior of captured compounds within the nanoparticle matrix is assessed using mean square displacement and diffusion coefficients. Compounds with high capture levels exhibit limited mobility, indicative of effective absorption and adsorption. Intermolecular interaction analysis highlights the significance of hydrogen bonds and electrostatic interactions in the pesticide-polymer association. Notably, two promising candidates, an antibiotic derived from tetracycline and a rodenticide, demonstrate a strong affinity for CAC nanoparticles. This computational methodology offers a reliable and efficient screening approach for identifying effective pesticide capture agents, contributing to the development of eco-friendly strategies for pesticide removal.

## 1. Introduction

Pesticides are a class of poisonous chemicals intentionally used in agriculture, forestry, public health, and homes to control pests and increase crop yields. They can be categorized into various types based on their target organisms and chemical properties. For instance, herbicides are used to control weeds, insecticides are used to kill insects that can cause significant crop damage and transmit diseases; fungi are controlled through fungicides [1,2,3]. Although pesticides can be effective in controlling pests, they also have the potential to cause harm to non-target organisms and the environment [4]. Pesticides can persist in the environment for long periods, accumulate in the food chain, and have adverse effects on beneficial insects [5], birds [6,7], and mammals [8,9]. In addition, pesticide residues in food and water can also pose health risks to humans, such as developmental and neurological disorders, cancer, allergies, and asthma among other chronic diseases [2,10,11,12,13]. Approximately 7000 individuals worldwide die due to unintentional poisonings annually, with these incidents being closely linked to excessive exposure and unsuitable usage of poisonous substances [14]. To minimize the negative impacts of pesticides, it is essential to adopt sustainable pest management practices, which aim to reduce pesticide use and promote the use of alternative pest control strategies, such as crop rotation [15], natural predators [16], and resistant crop varieties [17]. Governments also play a critical role in monitoring and regulating pesticide use to ensure that they are used safely and effectively. However, these measures do not solve the immediate problem, i.e., the presence and accumulation of pesticides in different environments such as aqueducts, rivers, lakes, and other freshwater sources. In this sense, bio-nanotechnology provides us with strategies to adsorb and entrap pesticides within polymer nanoparticles [18,19,20,21,22]. The biosorption process refers to the unique ability of certain biomolecules or polymeric matrices to effectively capture and accumulate specific ions or molecules from water-based solutions. This natural phenomenon occurs when functional groups present on the surface of biomaterials interact with the target substances in the solution [23]. To provide further clarity, it is important to understand the related processes of adsorption and absorption. Adsorption refers to the adhesion of molecules or ions onto a surface, typically without penetration or diffusion into the adsorbent material. On the other hand, absorption involves the penetration and diffusion of substances into the bulk of a material.

Polyelectrolyte complexes are becoming increasingly important materials due to their ability to encapsulate various substances [24,25]. The high surface area of polyelectrolyte complexes, combined with their ability to selectively bind to specific molecules, makes them an excellent candidate for capturing pesticides from water sources. The high entropy associated with the release of counter-ions and the strong electrostatic attraction between oppositely charged polymers leads to an attractive polymeric matrix solution for molecule capture [26]. Chitosan is a biopolymer that has gained significant attention due to its numerous advantages as a versatile biomaterial. It is derived from chitin, which is a naturally occurring polysaccharide found in the shells of crustaceans such as shrimp, crab, and lobster [27]. Chitosan possesses several unique properties, including biodegradability, biocompatibility, and antimicrobial activity [28,29,30]. The chemical structure of chitosan consists of a linear chain of β-(1→4)-linked D-glucosamine and N-acetyl-D-glucosamine units. One of the major advantages of chitosan is its cationic nature (cationic polyelectrolyte), which allows it to interact with negatively charged biomolecules such as the alginate matrix [31,32,33]. The residue positive charges in the former are due to primary functional amine groups (-NH_2_) which are some of the most important functional groups in chitosan chemistry. The amine group on chitosan provides a reactive site for the conjugation of various biomolecules, such as proteins, peptides, and drugs [34]. Alginate (anionic polyelectrolyte) is a naturally occurring polysaccharide obtained from brown seaweeds, such as *Laminaria hyperborea*, *Ascophyllum nodosum*, and *Macrocystis pyrifera*. One of the major advantages of alginate is its ability to form hydrogels in the presence of divalent cations, such as Ba^2+^, Ca^2+^, Sr^2+^, Cu^2+^, Zn^2+^, Ni^2+^, and Mn^2+^ [35,36]. The chemical structure of alginate consists of a linear chain of β-(1→4)-linked D-mannuronic acid (M) and L-guluronic acid (G) residues [37,38,39,40]. These natural biopolymers have a wide range of applications in various fields such as food, pharmaceuticals, and biomedicine. Their potential use has gained significant attention due to their unique properties and versatility. However, the use of these biopolymers is subject to regulations in certain applications. For instance, in the United States, the FDA regulates the use of alginate and chitosan as food additives and in pharmaceutical products. These biopolymers are generally recognized as safe for use in these and other applications and are subject to regulations regarding safety, efficacy, and quality as outlined by the FDA and the EMA. For these reasons, their unique properties and versatility have led to increased interest in their potential use as biosorption systems and bioremediation [41]. Alginate and chitosan, either together or separately, have proven to be a valuable solution for the adsorption of compounds, whose accumulation is highly harmful to the environment and human health, among these we find heavy metals [42,43,44,45,46], dyes used in the food industry [47,48,49,50] and pesticides [51,52,53,54,55,56]. However, to the best of our knowledge, there is no exhaustive theoretical study to date that has tested a calcium-alginate-chitosan (CAC) nanoparticle against a wide range of pesticides.

The aim of this work was to evaluate the affinity and adsorption-absorption competence of a CAC nanoparticle against a pesticide database using a theoretical-computational approach such as ensembled-based virtual screening, Molecular Dynamics simulations (MDs) and quantum calculations. At the same time, this study attempts to provide a workflow for the rapid identification of the best candidates interacting with polyelectrolyte nanoparticles.

## 2. Materials and Methods

### 2.1. Pesticide Database Preparation

A Registered Pesticides of Aquatic and Ecological Life library with 624 compounds (https://www.epa.gov/pesticide-science-and-assessing-pesticide-risks/aquatic-life-benchmarks-and-ecological-risk, accessed on 20 February 2023) from the United States Environmental Protection Agency (EPA) was used for ensemble docking. The library was prepared as follows:

- We only considered compounds that were registered with the Chemical Abstracts Service (CAS).
- To conduct a comprehensive search, we utilized the PubChem database [57] and selected the advanced search option to filter compounds by their unique CAS identifier.
- Using the RDKit python library (RDKit: Open-source cheminformatics. https://www.rdkit.org, accessed on 3 March 2023), we processed all compounds in the final database by generating 100 conformers per molecule. Each conformer was evaluated using the ETKDGv3 conformer generator [58], a stochastic search method that combines distance geometry with knowledge obtained from experimental crystal structures.
- To maintain uniformity in our compound preparations, we employed OpenBabel software [59] to adjust the pH of all samples to a range of 7.0 ± 2.0.

The compound library is available in a .csv database format, with each compound listed by CID identifier, IUPAC name, SMILES notation, and their respective physicochemical properties.

### 2.2. Calcium-Alginate-Chitosan Nanoparticle Preparation

**Figure 1:**
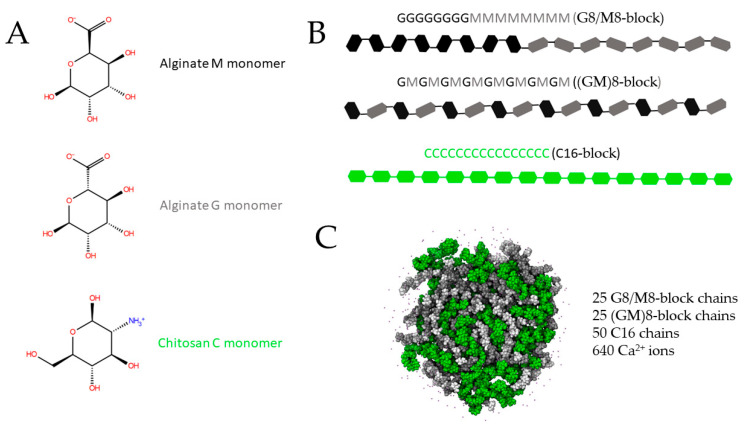
Nanoparticle preparation. (**A**) monomers from alginate and chitosan showing their charged groups at pH 7.0. (**B**) chains formations for alginate and chitosan. (**C**) nanoparticle built into a sphere of 70.0 Å radius. The number of chains of alginate and chitosan and the calcium ions are described in (**C**). The M and G alginate monomers are depicted in light and dark gray, and chitosan monomers are shown in green color.

Previously, we showed that the alginate monomers: M and G, have an acid dissociation value (pKa) of approximately 3.6 in their carboxylic acid and that above this value they have a higher absorption potential as they possess higher porosities [40]. In consequence, we employed the same configuration previously reported. The pKa value of ca. 6.3 for chitosan monomer (C) was predicted with Epik software [60] and corroborated with the literature, being a molecule protonated at its amino group at pH 7.0 (pH considered in this work). The monomers used in this study and their charged atoms are depicted in Figure 1A. The M and G subunits served as initial points to construct two alginate chains, comprising 16 monomers with an equal ratio of M and G units. One chain was created by joining eight consecutive G subunits with eight M subunits, forming a G8/M8-block pattern, while the other chain was composed of alternating G and M pairs, resulting in a (GM)8-block pattern, as illustrated in Figure 1B. Additionally, a chitosan chain was formed by linking 16 C monomers named (C16). The chains C16, G8/M8-block, and (GM)8-block were minimized by molecular mechanics by using the steepest descent algorithm with OPLS3e as the force field [61] in an implicit solvent (VSGB 2.1 [62]), using the Maestro-Schrödinger suite [63]. The maximum number of iterations (5000) and the convergence threshold were the same as reported in [40]. The nanoparticle formed by a composition of calcium-alginate-chitosan (CAC) was ensembled by using the Packmol software [64] involving 50 alginate chains (25 G8/M8-block and 25 (GM)8-block), 50 chitosan chains (16C) and 640 calcium ions. The molecules belonging to the nanoparticle CAC were randomly placed into a sphere with a radius of 70.0 Å, adding a tolerance cutoff of 4.0 Å between molecules positioned in the cartesian plane (Figure 1C). Subsequently, the CAC nanoparticle was solvated in a cubic water box of SPC molecules (172 Å × 172 Å × 172 Å), adding chloride as a counterion to neutralize the system. We ran an Isothermal–Isobaric ensemble NPT (P = 1 atm and T = 300 K) Molecular Dynamics simulation (MDs) by 100 ns using the Desmond/Maestro-Schrödinger suite, OPLS3e as a force field, and the default relaxation protocol. Once the simulation was finished, the thermodynamic stability of the nanoparticle was analyzed by computing the root-mean-square-deviation (RMSD) and the radius of gyration (Rg) to both selections; the alginate-chitosan and the alginate alone.

### 2.3. Ensemble Conformers Selection

We used the RMSD computed to the most stable part of the molecular trajectory as input to generate a dendrogram of 20 clusters of CAC nanoparticles through the Bio3D package in R [65]. Consequently, we randomly chose one frame of the trajectory *per* cluster, which was exported to PDBQT format for subsequent analysis.

### 2.4. Ensemble-Based Virtual Screening

It is commonly acknowledged that macromolecules such as CAC nanoparticles do not have a fixed structure in solution, but rather exist as a dynamic ensemble of conformations. Ensemble-docking enables us to test several conformations of the CAC nanoparticle against the pesticide database. For this, for each of the 20 previously obtained conformations, four grids were made around the surface of the polymer. Accordingly, the 80 grids were used to perform docking against the database comprising 624 pesticides with AutoDock Vina-GPU 2.0 [66]. Five metrics (Table 1) were employed to identify the best five candidates interacting with the nanostructure through the Vina docking energy: minimum value, arithmetic mean, geometric mean, harmonic mean, and median, like the fusion rules reported by Bajusz D. et al. [67]. The docking outputs were analyzed in an in-house R script available at: https://github.com/chedgala/VINA-GPU_parser, accessed on 10 March 2023. Our script ranks each of these five metrics from best to worst score (where the most negative energy is considered the best value) by pesticide, and then we select those pesticides that meet two consensus criteria: (1) those pesticides that have a statistic mode a ranking in the first five positions and (2) that this mode is found in at least three of the five metrics evaluated.

### 2.5. Pesticides Capture Studies

We measured and ranked the molecular capture of each of the best five pesticide candidates (here termed CA1, CA2, CA3, CA4, and CA5) entering the CAC nanoparticle over time. For this, (I) since CA1, CA4, and CA5 have ionizable groups whose pKas are reasonably near to the pH value of this study (7.0), we consider their two forms: charged and neutral for CA1 and CA4 and the charged form for CA5, independently for the subsequent analysis. (II) We built a new CAC nanoparticle in a smaller sphere of 40 Å radius, which contained 10 G8/M8-block chains, 10 (GM)8-block chains, 20 chitosan chains and 320 calcium ions. This system was used as a starting point for adding the candidates in the next step. (III) For each candidate (and charge state if applicable), 10 copies were generated and positioned inside a sphere of 80 Å radius and outside another sphere of 70 Å. Therefore, there is a buffer region of 30 Å separating the pesticides from the CAC nanoparticle. These seven systems: CA1_charged, CA1_neutral, CA2_neutral, CA3_neutral, CA4_charged, CA4_neutral, and CA5_charged were solvated, neutralized, and simulated by 100 ns in triplicate *per* system, in the same way as previously described for the CAC nanoparticle alone, totalizing a simulation time of 2.1 µs. In order to identify the pesticides that are captured by the nanoparticle, we quantified the number of candidates that were at a distance ≤ 3.5 Å away from the CAC nanoparticle in each time step. Besides, we computed a parametric Student’s *t*-test comparing against the best candidate.

### 2.6. Non-Covalent Interactions

For every representative conformation (obtained from a 100 ns simulation) identified through cluster analysis, the non-covalent interaction index (NCI) [68] was determined. Non-covalent interactions, which include hydrogen bonds, steric repulsion, and van der Waals interactions, were identified and mapped by analyzing the promolecular densities (*ρ^pro^*). These densities were calculated by adding up the atomic contributions. The NCI takes into account the electron density (*ρ*), its derivatives, and the reduced density gradients (*s*).

(1) $$s=\frac{1}{2{\left({3\pi }^{2}\right)}^{1/3}}\;\frac{\nabla \rho }{\rho^{4/3}}$$

These interactions occur in a specific area and are visible in the form of low-gradient isosurfaces with low densities in real space. The isosurfaces are interpreted and colored based on the corresponding values of the sign *ρ*(*λ*_2_). The colors used for the surfaces follow a blue-green-red scale which reflects the strength and type of interaction. Blue represents strong attractive interactions, green represents weak van der Waals (vdW) interactions, and red represents strong unbound superposition.

### 2.7. Adsorbent and Absorbent Characterization

Finally, to quantify the absorption/adsorption capacity of CAC nanoparticles for each previously simulated candidate, three descriptors were computed throughout the simulation time. The first descriptor aimed to identify the relative position of the candidate within the CAC nanoparticle by analyzing the density profile. This allowed us to determine whether the candidate was absorbed, adsorbed, or remained outside the nanoparticle. We compute the linear projection of the mass density profile along the Z-coordinate for all candidates in each time step by means of the VMD Density Profile Tool [69]. For this, the axis was divided into 240 windows (from −120 to 120 Å) and the density was calculated *per* each window and averaged for the whole time (100 ns). For more details see the publication Giorgino [69].

The second descriptor involved assessing the relative displacement of each candidate within the water-box system. This analysis provided insights into the mobility and diffusion behavior of the candidates throughout the simulation trajectory. By calculating diffusion coefficients (DC) and mean square displacements (MSD), we could quantify the degree of movement and explore how each candidate interacted with the nanoparticle matrix. DC and MSD were calculated through the Diffusion Coefficient Tool [70] in VMD [71].

The third descriptor focused on the number of intermolecular interactions between each candidate and the polymer matrix, specifically hydrogen bonds (HBs) and salt bridges (SBs) interactions. These interactions play a crucial role in determining the adsorption/absorption behavior and the stability of the candidate-polymer complex. Both types of interactions were analyzed in Desmond/Maestro-Schrödinger suite.

## 3. Results and Discussions

### 3.1. Pesticide Database Characterization

In this study, we examined two sets of standards in the US: the Aquatic Life Benchmarks for freshwater species from the Office of Pesticide Programs and the Ambient Water Quality Criteria for registered pesticides from the Office of Water. These benchmarks are used together with environmental chemistry methods (ECMs) to detect pesticide residues in soil or water. The benchmarks are based on scientific studies reviewed by the Environmental Protection Agency (EPA) to evaluate the potential harm to freshwater organisms caused by exposure to pesticides and their byproducts in recent ecological risk assessments.

**Figure 2:**
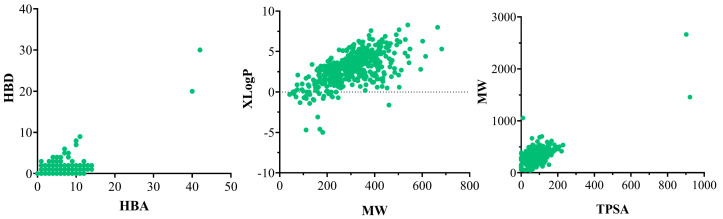
Distribution of pesticide database in terms of the (**left**) number of HBA and HBD, (**center**) LogP and MW, and (**right**) MW and TPSA. Each green dot represents the pesticides evaluated.

We observed that the average and 90th percentile of physicochemical properties of pesticides (Table 2), such as molecular weight (MW) is less than 500 Da, meaning that they may be more prone to volatilization and leaching, which may result in their dispersion in the environment and contamination of water and air. Topological polar surface area (TPSA) values of the species are high (greater than 50 Å^2^), which makes them more soluble in water and other polar solvents, which may facilitate their dispersion and transport in the environment and water pollution. In the case of the partition coefficient (LogP), logP values are high, indicating higher hydrophilicity, are more soluble in water, and thus are more mobile and bio-accessible to aquatic organisms. These pesticides may have a higher potential for water pollution, especially in water bodies with high renewal and circulation rates. Figure 2 shows the correlation between the number of HBA and HBD. From this graph, we observe that most of the pesticides present up to 7 HBA and up to 12 HBD. In addition, the correlation between MW and lipophilicity (LogP) of the pesticides is shown. From this analysis, we observe that most of the species are distributed in a range of MW up to 500 Da, and a LogP between −2.0 and 6.0. The solubility in water by the polar interactions of pesticides is an undesirable property, because of the pollution they cause and has a negative impact on marine biodiversity; therefore, we observed that the correlation between the MW and the polar surface of pesticides presents an MW range of up to 500 Da and a TPSA between 9 and 157 Å^2^. It is important to note that these physicochemical properties of a pesticide are not necessarily related to its toxicity. The efficacy of a pesticide depends on its chemical structure, its mode of action and its formulation.

It is evident that there is a greater emphasis on the synthesis of pesticides by academic and industrial research groups, as compared to research focused on capturing these chemicals. This trend has resulted in a steady increase in the number of pesticides being used as agrochemical agents, which in turn requires the development of innovative strategies for their removal from contaminated water sources. In this context, researchers have been exploring the potential of polymeric matrices as a viable solution for capturing these chemicals and enabling effective water decontamination.

### 3.2. Nanoparticle Formation

**Figure 3:**
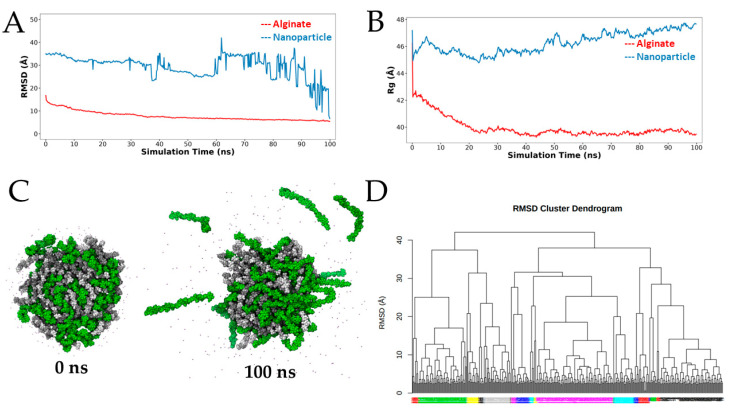
Nanoparticle compaction. (**A**) Root-mean-square-deviation (RMSD) and (**B**) Radius of gyration (Rg) of whole nanoparticle (alginate and chitosan chains) and alginate during the entire simulation time. (**C**) First (0 ns) and last (100 ns) frames of the simulation. G and M monomers are depicted in light and dark gray and C monomers in green color, the Ca^2+^ ions are depicted as points. (**D**) Cluster dendrogram calculated from the RMSD values, the 20 clusters are depicted in different non-consecutive colors.

The RMSD is commonly employed in molecular dynamics simulations of polymers to quantify the degree of structural changes and compactness during the time. Here, we analyze the RMSD by comparing the positions of the atoms at each time step to the initial conformation. The RMDS was computed for the whole nanoparticle (alginate and chitosan) and for the alginate chains alone, both depicted in Figure 3A. If we consider the complete nanoparticle, we can observe long fluctuations in the RMSD profile after 60 ns, which is indicative of thermodynamic instability. However, when we evaluate the RMSD only for the alginate chains, we can observe that the profile drops to around 25 Å considering the first 90 ns of simulations. We note the same differences when analyzing the Rg (Figure 3B). In this case, the Rg is a measure of the spatial distribution of the polymer’s mass around its center of mass and it is useful for identifying changes in the polymer’s conformation or compactness during the simulation. During the initial conformation, the Rg was similar for the nanoparticle (Rg^nanoparticle^) as for the alginate chains (Rg^alginate^) with a value of 47 Å, approximately. However, at the beginning of the simulation, the Rg^alginate^ decreased by almost 4 Å, and then gradually decreased until stabilizing at around 40 Å after 20 ns. On the other hand, we can observe in Rg^nanoparticle^, which begins to increase from 60 ns, reaching a maximum value at the end of the simulation of approximately 48 Å. Both metrics (RMSD and Rg) describe inadequate compactness of the complete CAC nanoparticle. However, if we visually inspect the simulation trajectory, as we represent in Figure 3C for the first and last ns, we find that this poor compactness is due to only four chitosan chains that did not properly bind to the rest of the nanoparticle. These chains represent only 8% of the chitosan chains or 4% of the total simulated chains. All the other chains (50 alginate chains and 46 chitosan chains) achieved proper compactness, similar to what we have reported in previous studies [51]. We took 20 conformational states of the CAC nanoparticle from the segment of the trajectory after 20 ns, taking into account that compactness, RMSD and Rg profiles of alginate stabilized after that time. These conformational states were obtained from a dendrogram formed with RMSD values (Figure 3D).

### 3.3. Virtual Screening Analysis

**Figure 4:**
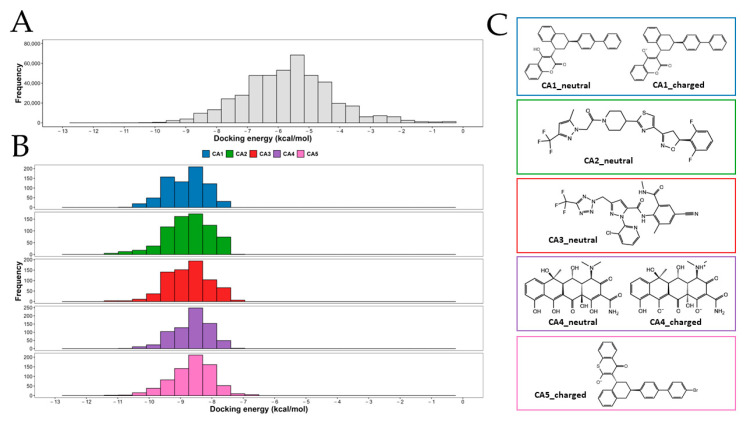

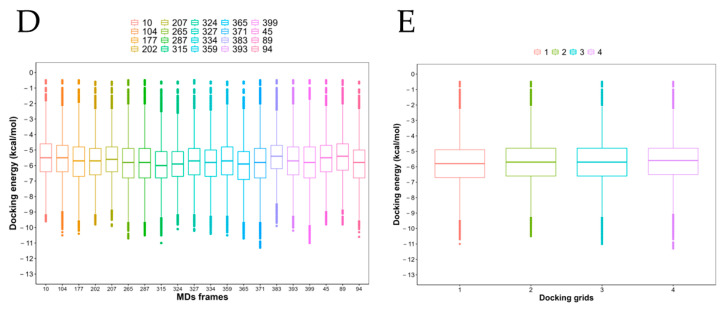
Docking energy distribution and best pesticide candidates: The Vina docking energy obtained for the whole database of pesticides (**A**) and for the best five candidates interacting with CAC nanoparticles (**B**). The ionized states for each candidate are depicted in (**C**). Docking energy variations through (**D**) 20 conformational states and (**E**) four docking grids.

The 264 pesticides and their respective ionized forms were docked into the 20 conformational states of the CAC nanoparticle through four docking grids per conformation. Therefore, 80 docking runs for each pesticide with at most nine docking poses totalizing 449,280 docking evaluations with Autodock Vina-GPU 2.0 software were performed and analyzed. In Figure 4A, we present the docking energy histogram along the whole pesticide database. The median and mean docking energy are −5.70 kcal/mol and −5.68 kcal/mol, while the first and third quartiles are −6.60 kcal/mol and −4.80 kcal/mol, respectively; 80% of docking poses are distributed within the range of −7.6 kcal/mol to −4.4 kcal/mol. We identified 2371 and 5502 outliers with the best and worst docking energies compared to the rest of the pool (data not shown but accessible through our script and files). In general terms, the docking energy distribution is uniform for the 20 conformational states derived from the MDs and for the four grids evaluated (Figure 4D,E). We wrote an R-script that parses the Vina-GPU outputs and ranks the whole database based on different statistical criteria, such as geometric, harmonic, or arithmetic mean, median and minimum value (best score). Each metric has its own ranking. Subsequently, these metrics were sorted to identify which pesticides ranked top in at least three of five metrics (Supplementary Figure S1 and Figure S2). The best candidates were: 54,676,884, 56,945,145, 56,602,311, 54,675,779, 54,680,782, and 91,771 according to their PubChem identifiers. However, 54,675,779 and 54,680,782 belong to the same pesticide in another tautomeric form. Consequently, we selected only one isomer for the subsequent analysis. Each of the selected pesticides was labeled with the acronym CA (*per* candidate) followed by the number used in the overall ranking: 54,676,884 (CA1), 56,945,145 (CA2), 56,602,311 (CA3), 54,680,782 (CA4) and 91,771 (CA5). Figure 4B shows the docking energy histogram for each candidate selected, which can also be compared to the histogram in Figure 4A since they have the same docking energy scale. We found that the best five candidates are distributed in the first quartile, with energy values between −11.30 kcal/mol to −6.80 kcal/mol and a median between −8.70 kcal/mol to −8.60 kcal/mol.

CA1 candidate (id: 54676884) corresponds to a difenacoum (2-hydroxy-3-[3-(4-phenylphenyl)-1-tetralinyl]-4- chromenone) is an antivitamin K anticoagulant of the second generation. It is used as a rodenticide in rodent pest control in the agricultural industry and it is efficient for the control of warfarin-resistant rodents [72]. Difenacoum causes primary poisoning of domestic animals and indirect poisoning of wildlife when they consume contaminated rodents. Its application in open areas is not recommended due to its ecotoxicity. It has also been reported that difenacoum shows persistence in tissues, which could cause death in exposed species [73]; therefore, this compound has a high ecotoxicity.

CA2 candidate (id: 56945145) is a fungicide named oxathiapiprolin (1-(4-(4-(5-(2,6-difluorophenyl)-4,5-dihydro-3isoxazolyl)-2-thiazolyl)-1-piperidinyl)-2-(5-methyl-3-(trifluoromethyl)1H-pyrazol-1-yl)ethanone), a potent piperidinyl thiazole isoxazoline inhibitor for oomycetes widely used as a fungicide in the agricultural industry. Acute toxicity tests of oxathiapiprolin in aquatic organisms have shown its toxicity in algae [74]. R-oxathiapiprolin, one of its three enantiomers, has 2.1 times higher toxicity in aquatic plants. Oxathiapiprolin has also shown toxicity at different stages of development in zebrafish [75].

CA3 candidate (id: 56602311) is a new anthranilic diamide insecticide named tetraniliprole developed by Bayer Crop Science for use on corn seed for the control of Lepidoptera, Coleoptera and Diptera insects. Tetraniliprole binds to the ryanodine receptor, activates the calcium channels of this receptor, and causes the outflow of intracellular calcium ions. This results in the death of the insect due to muscle contraction and paralysis [76]. Toxicity studies in earthworms have shown that cyantraniliprole and its metabolite could produce oxidative stress and subsequent cell damage in these annelids at a concentration of 5.0 mg/kg [77]. This shows that not only the insecticide produces ecotoxicity in soil, but also its metabolites.

CA4 candidate (id: 54680782) corresponds to oxytetracycline hydrochloride (OTC), a low-cost antibiotic derived from tetracycline used in livestock health and aquaculture [78]. Intensive use of OTCs could lead to antibiotic resistance problems in people and even lead to kidney disease and cancer. Because these chemical compounds accumulate in soil, water, and food [79]. Therefore, pollution caused by antibiotic use is an environmental problem of global magnitude.

CA5 candidate (id: 91771): is a second-generation anticoagulant rodenticide named difethialone, more toxic than the previous generation and with a prolonged persistence in tissues. The major problem with the use of this compound is that it produces secondary intoxication in other animals. Domestic and wild animals may repeatedly eat contaminated rodents, causing poisoning [80].

### 3.4. Capture Analysis

**Figure 5:**
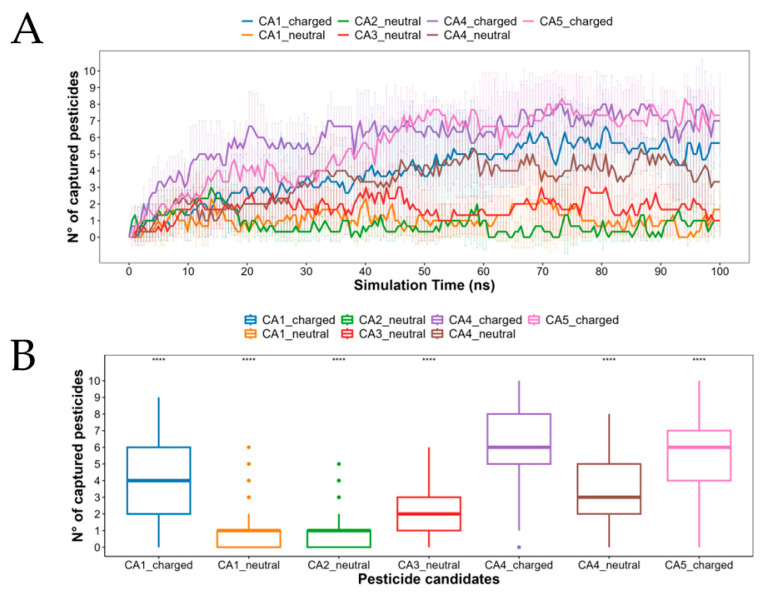
Capture of pesticides. The number of pesticides for each candidate (and their ionized states) at a distance ≤ 3.5 Å from the CAC nanoparticle (**A**) along the simulation time (mean ± s.d.) and (**B**) by a statistical analysis of a Student’s *t*-test comparing against the best candidate. **** = *p*-value ≤ 0.0001.

Calcium-Alginate-Chitosan hydrogels can form interstitial spaces, which are spaces between the polymer chains where water or other small molecules can diffuse. These interstitial spaces are important for the transporting of nutrients, oxygen, and other biomolecules into and out of the hydrogel. In this sense, intending to evaluate the capture capacity of the CAC nanoparticle for each pesticide at the molecular level, we run seven MDs by 100 ns (in triplicate) considering the ionized states for the best five candidates shown in Figure 4C. In general terms, the candidates with charged states (*q* = −1) such as CA1_charged, CA4_charged and CA5_charged were more rapidly captured within the CAC nanoparticle than those whose molecules with 0 net charge (CA1_neutral, CA2_neutral, CA3_neutral and CA4_neutral) as is represented in Figure 5A. The candidate CA4_charged showed on average the fastest capture achieving half of the simulated pesticides in contact with the polymer at 11.88 ns. The maximum percentage of pesticides captured was 80% reached by CA4_charged and CA5_charged at approximately 70 ns, as the mean in three replicas. The CA1_charged was, on average, better than CA4_neutral with a mean and maximum capture of 3.93 and 6.67 for CA1_charged and 3.35 and 5.33 for CA4_neutral, with a more pronounced difference after 60 ns. We performed a Student’s *t*-test to compare the whole group against the best candidate (CA4_charged). Our analysis shows a significant difference between the compared pesticides, with *n* = 606 observations *per* system (Figure 5B). When we analyzed each replica separately, we found that at least in one replica both: CA4_charged and CA5_charged reached 100% of pesticides captured, with a median of 6 in both cases. It is important to mention that both CA1 and CA4 were tested in their two protonation states, which according to our analysis can coexist at a pH of 7. However, CA1_neutral did not show an effective affinity for the CAC nanoparticle with a median of one and a maximum of six pesticides captured in one of three replicas, being it a poor candidate overall. A similar situation occurs with CA2_neutral and CA3_neutral, with a median of one and two pesticides captured, respectively.

### 3.5. Non-Covalent Interactions Analysis

**Figure 6:**
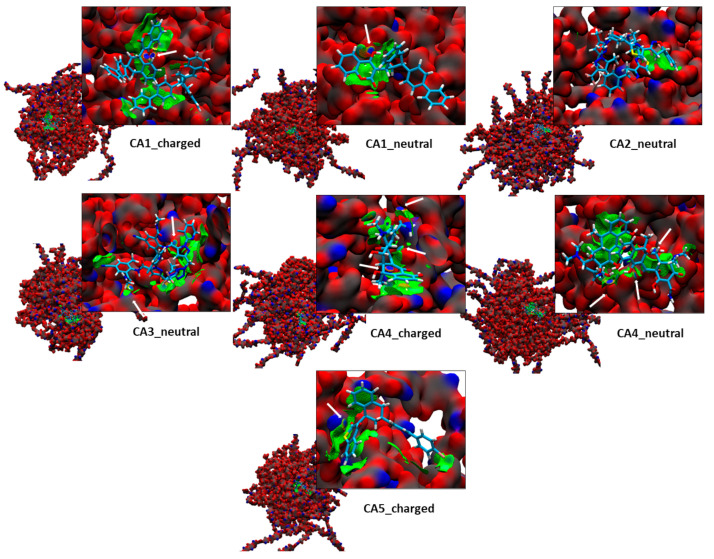
NCIplot isosurfaces (0.7 a.u) of the non-covalent interaction for the best pesticide candidates. Blue areas are strongly attractive, while green ones correspond to weak interactions. White arrows represent hydrogen bond interactions.

To complement and provide more details on the intermolecular interactions between the chosen pesticides and the CAC nanoparticle, an analysis of the NCI was carried out, using the promolecular electron density and the reduced density gradient, see Figure 6. The NCI present in the systems formed by the nanoparticle and pesticides (CA1_neutral, CA1_charged, CA2_neutral, CA3_neutral, CA4_neutral, CA4_charged, CA5_charged), it is evident that the attraction between the nanoparticle and pesticides are van der Waals type, π-π stacking and hydrogen bonds. In relation to the weak interactions, vdW type present in the systems, it is identified that for the pesticide CA1 in its neutral and charged form, the interaction is mainly dominated by the aromatic rings it has with the surface of the CAC nanoparticle, the same occurs for the case of CA2 and CA3 in its neutral form, where the fluorine and chlorine of aromatic rings and the trifluoromethyl group interact with the monomeric units of the nanoparticle, showing a weaker interaction. In the case of CA4 in its neutral and charged form, the interaction is mainly dominated by the polycyclic aromatic hydrocarbon group with the surface of the CAC nanoparticle. CA5 interaction is primarily dominated by the aromatic rings and terminal benzene bromine with the nanoparticle surface. All π-π stacking interactions occur when the aromatic rings of these pesticides are stacked parallel on the nanoparticle surface. These weak vdW interactions (green color, Figure 6) stabilize the capture and physisorption of pesticides on nanoparticles, where they are observed with higher isosurface between pesticides and polymers. The more pesticide molecules are added, the more they tend to localize on the nanoparticle’s surface.

In addition, we can see that the pesticides present electrostatic interactions as hydrogen bondss (blue color), which is a weaker interaction than a covalent bond, but stronger than van der Waals forces. The hydrogen bonds present in the systems are mainly identified as pesticides with hydroxyl groups, providing greater interaction and better capture of these molecules. In addition, it is appreciated that charged pesticides are better captured on a nanoparticle than neutral pesticides due to electrostatic attraction and the formation of hydrogen bonds.

### 3.6. Adsorption and Absorption Characterization

Identifying the mass density peaks in the density profile of a polymer is crucial for quantifying its absorption and/or adsorption capacities for a specific pesticide and understanding how they depend on the polymer’s physicochemical characteristics. These density peaks provide valuable information about the spatial distribution and localization of the pesticide within the polymer matrix. By analyzing the position and magnitude of these peaks, we can determine whether the pesticide is being absorbed into the polymer or adsorbed onto its surface. To quantify the mass density profiles, we utilize the Z-coordinate. In Figure S1H (in the Supplementary Information), we illustrate the relative localization of the CAC nanoparticle, which falls within the range of −30 to 30 Å. Focusing on the candidates that previously exhibited low levels of capture by the nanoparticle (CA1_neutral, CA2_neutral, and CA3_neutral), we observe that their density profiles within the polymer’s coordinate range (−30 to 30 Å) are mostly below 2 Da/Å^3^ in their three replicas (except for CA1_neutral replica 1), see Figure S1B–D. A similar trend is observed in the CA4_neutral system, where the highest density peaks are found beyond 30 Å, indicating a limited level of absorption by the polymeric matrix (Figure S1F). In contrast, the CA5_charged system exhibits density peaks exceeding 3 Da/Å^3^ from the nanoparticle surface to a depth of approximately 15 Å (Figure S1G), indicating significant adsorption and absorption levels. Likewise, the CA4_charged system displays an even distribution of density peaks exceeding 2 Da/Å^3^ from the surface to the interior of the polymer (Figure S1E). Among the evaluated candidates, the CA1_charged system consistently exhibits the highest density levels within the depths of the polymer in all three replicates, suggesting the highest level of absorption (Figure S1A).

Determining the MSD and DC of compounds within a polymeric matrix holds great significance in quantifying the absorption and/or adsorption capacities of the polymer. These parameters provide essential information about the compound’s mobility and its ability to move within the polymer matrix. By analyzing the MSD, we can assess the average distance covered by the compounds over a specific time, which reflects their diffusion behavior. Additionally, the DC provides a quantitative measure of the rate at which the compounds spread throughout the polymer. These measurements are instrumental in evaluating the polymer’s efficacy in facilitating the absorption or adsorption of compounds. Moreover, by comparing the diffusion characteristics of different compounds, we can gain valuable insights into their interactions with the polymeric matrix, enabling us to optimize polymer-based systems and enhance their absorption and adsorption capabilities. After comparing the MDS and DC of all the candidates evaluated along the trajectories in their three replicates (Figure S2), it becomes evident that the CA5_charged system has the lowest mobility and diffusion values, respectively. Our statistical analysis reveals that there is no significant difference between this system and CA4_charged in terms of both MSD and DC. Both systems consistently demonstrate a high degree of capture by the polymer and effective absorption within the polymer matrix. Therefore, it is reasonable to expect these molecules to exhibit low mobility once they interact with the nanoparticle. Interestingly, despite being highly captured by the polymer, the CA1_charged system ranks third in terms of MSD and DC. This finding may partially explain why this system penetrates the polymer to a greater extent, as evidenced by its density profile.

By characterizing and quantifying key interactions such as HBs and SBs, we can determine the strength and specificity of the pesticide-polymer association. This knowledge allows us to understand the driving forces behind the absorption and/or adsorption processes. In this sense, in Figure S3, we observe that CA4_charged exhibits the highest number of intermolecular interactions (HBs and SBs) with the polymer, indicating a strong affinity. Following this, the neutral counterpart of CA4 (lacking SBs due to its uncharged nature) demonstrates a slightly reduced number of intermolecular interactions. Lastly, CA5_charged and CA1_charged exhibit a lower number of intermolecular interactions, suggesting a weaker association with the polymer compared to the CA4_charged system.

Based on the comprehensive analyses conducted in this computational study, oxytetracycline hydrochloride and difethialone emerged as the most favorable candidates for interacting with the CAC nanoparticle. Both tetracycline and oxytetracycline have been reported as compounds able to be captured by different compositions of alginate and chitosan. Previous research has demonstrated the capture potential of different alginate and chitosan compositions for tetracycline and oxytetracycline. For instance, Erdem et al. reported the effectiveness of halloysite nanotube-based composites synthesized with chitosan and alginate biopolymers in removing tetracycline from wastewater [81]. Zang et al. employed an innovative solution of alginate-based nanofibers loaded with ZnO nanoparticles to efficiently absorb tetracycline, oxytetracycline, and doxycycline [82]. Furthermore, Zao et al. combined metal-organic frameworks (MOFs) with chitosan matrices to enhance the adsorption capabilities of single MOFs for tetracycline. Their density functional theory studies described intermolecular interactions similar to those presented in our analysis. Notably, to the best of our knowledge, no in vitro studies have reported the sorption of difethialone by alginate-chitosan compositions [82].

## 4. Conclusions

It is necessary to develop new and reliable methods to quickly test several toxic pesticides against a nanoparticle system to identify promissory candidates to be captured from the environment. The efficient capture of these compounds could help to maintain healthy ecosystems and the subsistence of aquatic and terrestrial organisms. In addition, these strategies would produce benefits for human health and the environment concerning the problem of the accumulation of these toxic compounds and their metabolites in nature. Here, we propose a computational methodology capable of testing several hundred pesticides in a specific nanoparticle (Calcium-Alginate-Chitosan, in this case), which allows us to identify two significant aspects when selecting the best pesticide capture agents: (1) the affinity of the toxic compound by the nanoparticle under study, and (2) the sorption ability of the nanoparticle to capture above-mentioned candidates in a short time. These aspects should be considered together in any initial study to avoid possible biases.

Furthermore, this study highlights the potential of CAC nanoparticles for removing pesticides from contaminated environments, and for future water treatment and agriculture applications. Here, we found two promising candidates interacting with CAC nanoparticles, where we show how the ionized state of the pesticide molecule affects its capture efficiency, with charged molecules being more rapidly captured within the polyelectrolyte CAC nanoparticle than neutral molecules. These candidates correspond to an antibiotic derived from tetracycline and a rodenticide, which interacts with the nanoparticle mainly by hydrogen bonds and electrostatic interactions.

The protocol proposed here is a promising alternative to capture toxic pesticides; however, we know that most of the current progress in this area is preserved at the laboratory level (wet lab). For this reason, it is necessary to optimize the conditions for its use on a large scale. In this sense, the use of hybrid technologies could be explored, which combine biosorption with nanoparticles as proposed here, with other traditional processes.

## Figures and Tables

**Table 1:** Metrics used to select the best candidates.

Rule	Formula
Minimum value	$min\;({DE}_{1},{\;DE}_{2}\ldots {DE}_{n})$
Arithmetic Mean	$\frac{1}{n}\sum_{i=1}^{n}{DE}_{i}$
Geometric Mean	$\sqrt[{n}]{\prod_{i=1}^{n}{DE}_{i}}$
Harmonic Mean	${\left(\frac{\sum_{i=1}^{n}{{DE}_{i}}^{-1}}{n}\right)}^{-1}$
Median	$median\;({DE}_{1},{\;DE}_{2}\ldots {DE}_{n})$

DE: Docking Energy denoted by ${DE}_{1},{DE}_{2}\ldots {DE}_{n})$.

**Table 2:** Calculated percentiles and statistical properties of main descriptors for pesticide database.

Descriptor	80th Percentile	90th Percentile	95th Percentile	Maximum	Minimum	Average
MW (Da)	408.4	447.2	503.3	2664.0	42.0	319.5
TPSA (Å^2^)	97.2	126.6	157.0	922.0	9.0	80.0
NRB ^[a]^	7	8	10	30	2	4
LogP	4.8	5.7	6.1	8.3	−5.0	3.0
HBA ^[b]^	7	9	10	42	0	5
HBD ^[c]^	2	2	3	30	0	1

^[a]^ Number of rotatable bonds. ^[b]^ Number of hydrogen bond acceptors. ^[c]^ Number of hydrogen bond donors.

## Data Availability

Not applicable.

## Supplementary Materials

The following supporting information can be downloaded at: https://www.mdpi.com/article/10.3390/polym15143020/s1. Figure S1: 1-D projection of mass density profiles depicted in (A) to (G) along the Z-coordinate for every candidate in three replicas computed for the whole simulation time. The CAC nanoparticle is distributed between −30 Å and 30 Å, approximately as is depicted in (H). Figure S2: Diffusion mobility for every candidate along the simulation time depicted by means of (A) the Mean Squared Displacement and (B) Diffusion coefficients. Both boxplots have embedded a table showing descriptive statistic for MSD and DC, such as mean, median, minimum, and maximum values. Both, MSD and DC were analyzed by a Student’s *t*-test comparing against the best candidate. ns = *p*-value > 0.005; * = *p*-value <= 0.05; ** = *p*-value ≤ 0.01; *** = *p*-value ≤ 0.001; **** = *p*-value ≤ 0.0001. Figure S3: Intermolecular interactions between CAC nanoparticle and every candidate type through A) hydrogen bonds and B) salt bridges along the whole simulation time. Both, HBs and SBs interactions were analyzed by a Student’s *t*-test comparing against the best candidate. ns = *p*-value > 0.005; * = *p*-value <= 0.05; ** = *p*-value ≤ 0.01; *** = *p*-value ≤ 0.001; **** = *p*-value ≤ 0.0001.
