# Supplementary material for: Calcium-Alginate-Chitosan Nanoparticle as a Potential Solution for Pesticide Removal, a Computational Approach
Source: Polymers (Basel). 2023 Jul 12;15(14):3020. doi: 10.3390/polym15143020 (PMC10383139; doi:10.3390/polym15143020)
Supplement: Supplementary file 1 [file polymers-15-03020-s001.zip › polymers-2450417-supplementary.pdf]

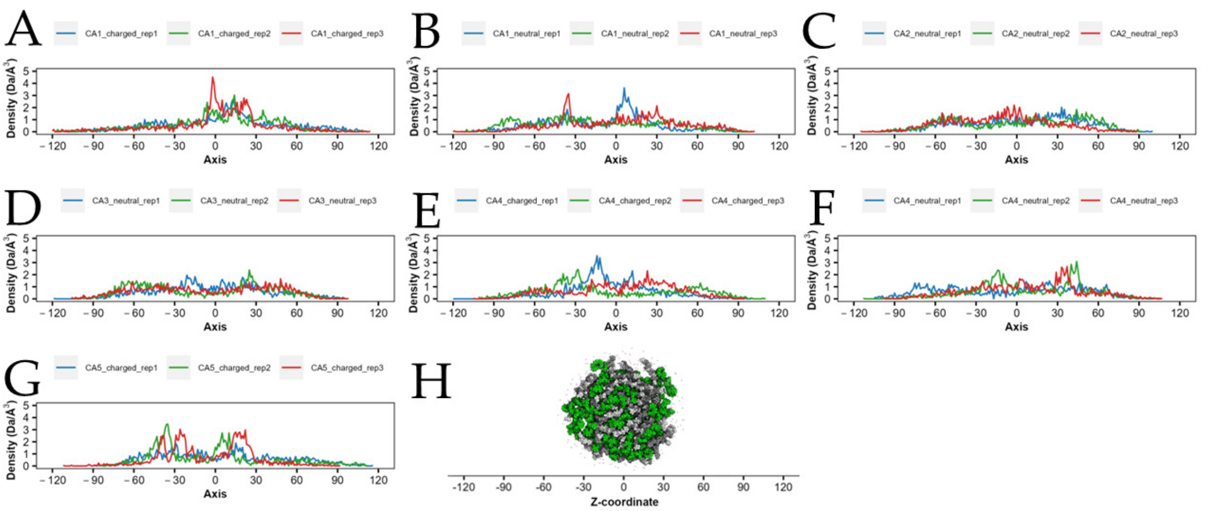

Figure S1: 1-D projection of mass density profiles depicted in (**A**) to (**G**) along the Z-coordinate for every candidate in three replicas computed for the whole simulation time. The CAC nanoparticle is distributed between −30 Å and 30 Å, approximately as is depicted in (**H**).

A

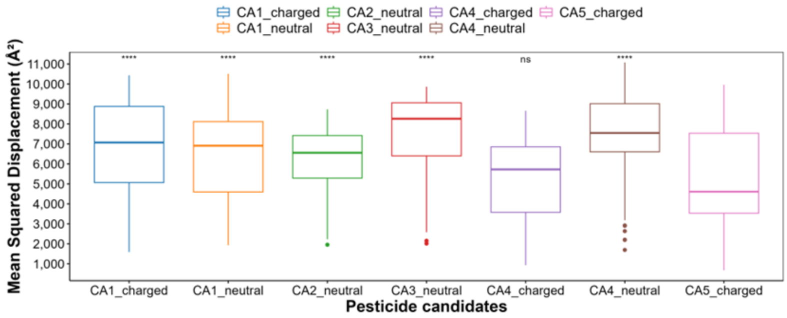

Mean Square Displacement (Å²)				
Candidate	Mean	Median	Min.	Max.
CA1_charged	6,946	7,451	2,058	8,019
CA1_neutral	6,632	6,959	2,296	7,566
CA2_neutral	6,242	6,605	2,173	7,487
CA3_neutral	7,667	8,193	2,248	8,823
CA4_charged	5,355	5,716	1,659	6,281
CA4_neutral	7,672	8,277	2,361	8,929
CA5_charged	5,301	5,670	1,450	6,368

B

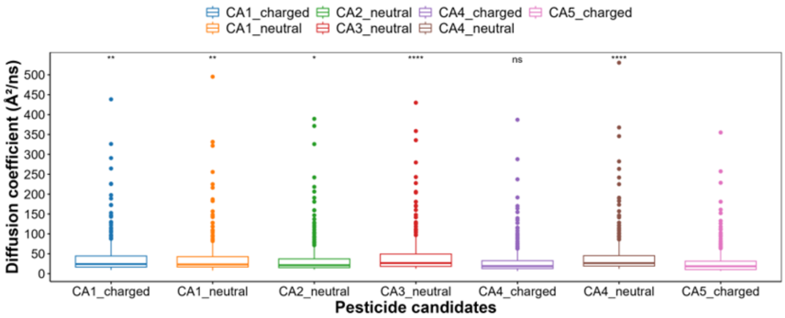

Diffusion coefficient (Å²/ns)				
Candidate	Mean	Median	Min.	Max.
CA1_charged	41.1	24.2	13.2	343.0
CA1_neutral	40.4	22.6	12.5	383.0
CA2_neutral	37.1	21.6	12.4	362.0
CA3_neutral	44.8	26.5	13.9	375.0
CA4_charged	31.1	18.6	9.81	277.0
CA4_neutral	44.9	27.1	14.6	393.0
CA5_charged	29.5	18.5	10.4	242.0
CA1_charged	41.1	24.2	13.2	343.0

Figure S2: Diffusion mobility for every candidate along the simulation time depicted by means of (**A**) the Mean Squared Displacement and (**B**) Diffusion coefficients. Both boxplots have embedded a table showing descriptive statistic for MSD and DC, such as mean, median, minimum, and maximum values. Both, MSD and DC were analyzed by a Student's t-test comparing against the best candidate. ns = p-value > 0.005; * = p-value <= 0.05; ** = p-value ≤ 0.01; *** = p-value ≤ 0.001; **** = p-value ≤ 0.0001.

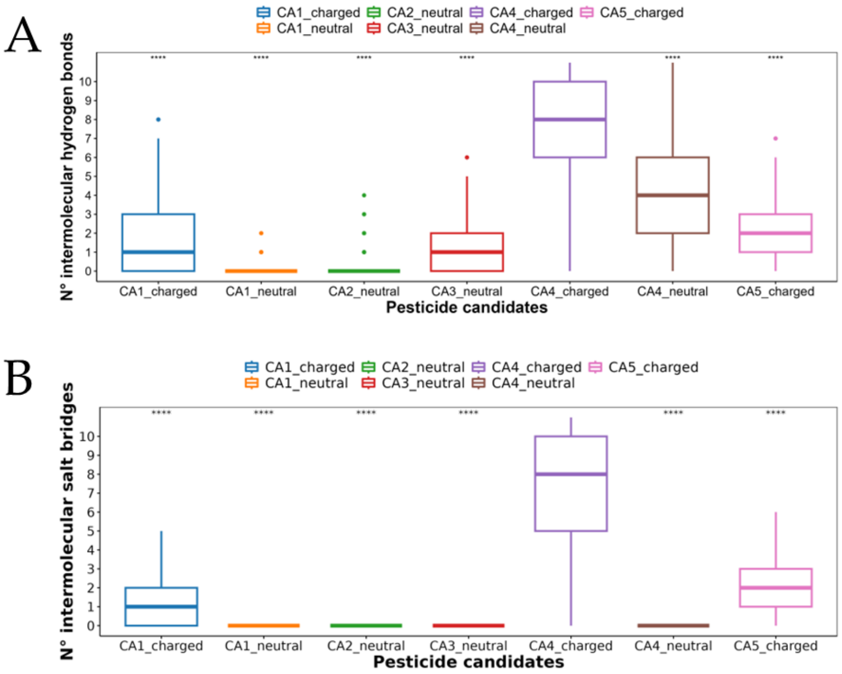

Figure S3: Intermolecular interactions between CAC nanoparticle and every candidate type through A) hydrogen bonds and B) salt bridges along the whole simulation time. Both, HBs and SBs interactions were analyzed by a Student's t-test comparing against the best candidate. ns = p-value > 0.005; * = p-value <= 0.05; ** = p-value ≤ 0.01; *** = p-value ≤ 0.001; **** = p-value ≤ 0.0001.
